# Distinguishing Dispositional and Cultivated Forms of Mindfulness: Item-Level Factor Analysis of Five-Facet Mindfulness Questionnaire and Construction of Short Inventory of Mindfulness Capability

**DOI:** 10.3389/fpsyg.2016.01348

**Published:** 2016-09-09

**Authors:** Wenjie Duan, Jinxia Li

**Affiliations:** Department of Sociology, Wuhan UniversityWuhan, Hubei, China

**Keywords:** factor structure, validity, mindfulness, short inventory of mindfulness capability

## Abstract

The widely used Five-Facet Mindfulness Questionnaire (FFMQ) mixes the dispositional and cultivated forms of mindfulness, thereby resulting in factor issues in previous studies. The present study distinguished the two forms of mindfulness and developed a Short Inventory of Mindfulness Capability at the item level of FFMQ. Three facets of mindfulness, namely, Describing, Acting with Awareness, and Non-judging of Experience, were assessed using community (*n* = 433) and student (*n* = 347) samples. Both meditators and non-meditators participated. Exploratory and confirmatory factor analysis (CFA) revealed a three-factor model of mindfulness with 12 items (four items per subscale). Psychometric evaluation demonstrated the solid factor structure of the measurement with high factor loadings, good internal consistency, and convergent validities. Longitudinal analysis indicated that the Acting with Awareness facet was a significant predictor of depression and anxiety symptoms 6 months later. Discussions focused on the roles of mindfulness capability on mental health as well as the relationship between them. A higher-order factor of mindfulness should be used to examine the efficacy of intervention or monitor the changes. Researchers who need to study the specific role or efficacy of each facet should calculate the scores of different facets.

## Introduction

Mindfulness initially appeared as therapy in clinical and medical fields (Kabat-Zinn, 1982). Meta-analysis studies have indicated that mindfulness-based approaches can significantly improve physical health of patients by reducing body-mind symptoms (Grossman et al., 2004; Ledesma and Kumano, 2009). Recently, mindfulness has been applied beyond clinical fields. Many studies have been completed on elderly care, workplaces, schools, and other settings, and they have focused on the relationships among positive mental health, well-being, and mindfulness training (Shapiro et al., 2008). Mindfulness-related interventions have been conducted in both clinical and non-clinical populations; such interventions have implied the significance of mindfulness to mental health in various contexts. The internal mechanisms of mindfulness in affecting well-being have been explored. Duan (2016) demonstrated that personal character strengths as the mediation between dispositional mindfulness and mental well-being. Accordingly, a reliable, valid, and efficient measurement for assessing mindfulness among various populations became necessary. The Five-Facet Mindfulness Questionnaire (FFMQ) may be the most widely used measurement (Park et al., 2013). Despite the extensive usage of FFMQ, the *post-hoc* conceptualization of mindfulness in the questionnaire has triggered a series of issues related to factor structure and validities.

Baer et al. (2006) combined the items of five inventories, namely, Mindfulness Attention Awareness Scale, Kentucky Inventory of Mindfulness Skills, Freiburg Mindfulness Inventory, Cognitive and Affective Mindfulness Scale-Revised, and Southampton Mindfulness Questionnaire, to form a large item pool (112 items). Exploratory factor analysis (EFA) revealed a 39-item five-facet structure (Baer et al., 2006), including Observing (noting both internal and external stimuli), Describing (using words to identify and express internal experience), Acting with Awareness (act-aware; paying full attention to the current activities without an automatic pilot), Non-judging of Experience (non-judging; non-evaluation of internal experiences; and stimuli), and Non-reactivity to Experience (non-reacting; no action toward emerging internal experience). FFMQ has been translated into Chinese (Deng et al., 2011; Hou et al., 2014), Swedish (Lilja et al., 2011), Italian (Giovannini et al., 2014), and French (Heeren et al., 2015), among others. The reliability and validity of FFMQ have been evaluated in various populations, including students (Deng et al., 2011; Lilja et al., 2011; Sugiura et al., 2012; Giovannini et al., 2014), teachers (Lilja et al., 2011), healthcare practitioners (Lilja et al., 2011), community adults (Giovannini et al., 2014; Hou et al., 2014; Williams et al., 2014), patients with fibromyalgia (Veehof et al., 2011), patients with psychological distress (Hou et al., 2014; Williams et al., 2014), and patients with mood and anxiety disorders (Curtiss and Klemanski, 2014). These increased findings from clinical and non-clinical areas not only preliminarily support the psychometric characteristics of FFMQ in measuring mindfulness in various populations but also questioned its factor structure.

Two major shortcomings have limited the understanding and application of FFMQ. First, the methodology to construct FFMQ mixes the dispositional and cultivated forms of mindfulness (Rau and Williams, 2016). For instance, the Mindfulness Attention Awareness Scale is considered a tool to measure traits (Mackillop and Anderson, 2007), whereas the Kentucky Inventory of Mindfulness Skills is used to assess mindfulness skills (Baer et al., 2004). Both theoretical and empirical evidence imply that dispositional and cultivated mindfulness are distinguished, and using the same inventory (e.g., FFMQ) to measure two constructs may result in controversial conclusions (Rau and Williams, 2016). Second, most previous studies (e.g., Baer et al., 2006; Deng et al., 2011; Curtiss and Klemanski, 2014; Hou et al., 2014; Williams et al., 2014) adopted item parceling to develop and evaluate the psychometric characteristics of FFMQ, which might have detrimental effects on models (Meade, 2006). The adoption of this method raises the question of whether all the items of a specific facet are highly and appropriately loaded on the corresponding latent factor. Moreover, whether all the items are closely related to the two essential facets of mindfulness is unclear, thus reflecting the content of present-centered awareness/attention and non-judgmental attitude. Given these two issues, existing results have suggested that the Non-react and Observing facets indicate unstable and weak psychometric properties.

The Non-react facet consistently exhibited low reliability (α < 0.50), test–retest reliability (*r* < 0.55), and factor loadings in previous studies (Deng et al., 2011; Veehof et al., 2011). Baer et al. (2006) found that the association of Non-react with overall mindfulness was extremely weak. The criteria-related correlation between Non-react and general severity index was also extremely low (*r* = −0.11) (Goldberg et al., 2016). Although these correlations improved after the four items of Non-react were rephrased (Baer et al., 2008; Veehof et al., 2011), Tran et al. (2013) demonstrated that the associations of Non-react and mental health across community and student samples remained inconsistent because these items were difficult to understand and were positively related to the suppression scale of Emotion Regulation Questionnaire. Bishop et al. (2004) strongly proposed the re-examination and revision of the Non-react items.

The Observing facet presents a serious issue. Initially, Baer et al. (2008) found that the relationship between this facet and psychological symptoms are only significant among meditated samples. They hypothesized that the Observing facet only exists in individuals with meditation experience; for those who without experience of mediation, such as students and community samples, the four-factor structure of mindfulness (without Observing factor) is the optimal choice (Baer et al., 2006, 2008). Recent studies have confirmed this conclusion. Williams et al. (2014) compared the factor structures among the non-meditated adults, meditated adults, and depressed adults with minimal meditation practice. Five comparable models were constructed, including a comprehensive mindfulness one-factor model (Model 1), five-factor related model (Model 2), hierarchical five-factor model (Model 3), four-factor related model (Model 4), and hierarchical four-factor model (Model 5). The results showed that the four-factor model was the best in non-meditated samples, whereas the five-factor model was the best in meditated samples (Williams et al., 2014).

Nevertheless, inconsistent findings were obtained in other studies. Rather than the four-factor model (Baer et al., 2006, 2008), Deng et al. (2011) obtained a five-factor related model, with an acceptable goodness-of-fit index among a non-meditated student sample. Hou et al. (2014) also validated that the correlated five-factor model was better than the hierarchical five-factor one in combined community (including both meditated and non-mediated participants) and non-meditated clinical samples. These inconsistent findings challenged the hypothesis that Observing was a unique factor that existed among meditators. A psychometric test further demonstrated that the differential item functioning of FFMQ between meditators and non-meditators with similar demographic characteristics is relatively small (Baer et al., 2010).

The divergent findings imply a need to re-explore the items and facets of mindfulness in FFMQ and distinguish the facets of dispositional and cultivated mindfulness. Mindfulness, as a basic human quality characterized by the present-centered awareness and non-judgmental attitude (Kabat-Zinn, 1990, 2003), should be shared by both meditators/individuals with meditation experience and non-meditators/individuals without meditation experience. Mindfulness should also be cultivated through intentional training and interventions. Two problematic facets, namely, Observing and Non-react, were hypothesized as dispositional facets, whereas Describing, Non-judging, and Act-aware were recognized as cultivated facets. The present study developed a short inventory to measure mindfulness capability. The evaluation at the item level ensured that all of the items were loaded to the overreaching factor, which precisely represented the construct. A community sample, which involved both meditators and non-meditators, was adopted to identify and clarify the closely related items of mindfulness. A college student sample was further used to confirm the factor structure and test the 6-month predictive ability.

## Methods

### Participants and procedures

The participants involved in this study came from four communities and two universities in China. The inclusion criteria were as follows: (1) 18 years old and above, (2) native language is Chinese, (3) in second or third year if a student, (4) can complete a paper-and-pencil questionnaire package within 20 min, and (5) willing to provide a cellular phone number and an email address for further communication. Participants with self-reported active physical and mental illnesses were excluded. Written informed consent was obtained before completing the questionnaire. The first data collection was conducted (Time 1 or T1) among the community and student populations. The second collection was administrated 6 months later (Time 2 or T2). The participants from communities were asked to complete FFMQ at T1, and the students were required to complete FFMQ, Flourishing Scale (FS), and Depression Anxiety Stress Scales-21 (DASS-21) at both T1 and T2. FS was more suitable for using among ordinary and health adults compared to Brief Inventory of Thriving (Duan et al., 2016). The Institutional Review Board of the Wuhan University approved this study.

The community sample comprised 433 adults (227 females and 206 males), whose ages ranged from 19 to 73 (*M* = 40.32, *SD* = 14.22). Among the sample, 69 completed primary education, 199 completed secondary education, and 165 completed higher education (i.e., undergraduate and postgraduate). More than half (*n* = 285) were married, and 148 were unmarried (i.e., single, divorced, and widowed). Among the participants, 201 reported meditation experience. The college student sample comprised 168 females and 179 males. The age range was 19–24 (*M* = 21.31, *SD* = 0.88). Among the participants, 284 were single and 63 were in a relationship. None of the participants reported a history of serious illness, long-term medication, and meditation experience.

### Measurements

#### FFMQ

The 39-item FFMQ was constructed to assess five facets of mindfulness, including Observing, Describing, Acting with Awareness, Non-judging of Experience, and Non-reactivity to Experience. The internal consistency of the FFMQ ranged from 0.67 to 0.93 (Baer et al., 2006). Participants were required to answer 39 items using a five-point Likert scale (1 = never or rarely true to 5 = very often or always true). Certain items have reverse scoring. A high mean score in each subscale reflects a high level of the different facets of mindfulness. The psychometric characteristics of the Chinese version of the FFMQ have been established well in previous studies (Deng et al., 2011; Hou et al., 2014).

#### FS

FS was used to measure the important aspects of psychological well-being of human beings via eight items (Diener et al., 2010), which include engagement, relationship, competence, and life purpose. The participants were required to provide individual answers according to a seven-point scale, that is, from strongly disagree (1) to strongly agree (7). Mean scoring was adopted. A high score denotes high function and success level of a respondent. Positive psychometric properties (e.g., univariate structure and high Cronbach's α) characterized the scale (Diener et al., 2010). The simplified Chinese version of the scale was validated using community and adolescent samples (Duan and Xie, 2016; Tang et al., 2016), which demonstrated satisfied reliabilities and validities. The Cronbach's alpha of the community sample is 0.82 and that of the student sample is 0.87.

#### DASS-21

DASS-21 is a short version of DASS for assessing Depression, Anxiety, and Stress over the past week; it is a 21-item self-reporting scale that contains three subscales (seven items per subscale) (Lovibond and Lovibond, 1995). The participants were asked to indicate individual answers on a four-point scale based on their experiences in the past week, that is, from “did not apply to me at all” (0) to “applied to me very much, or most of the time” (3). None of the items was reversely coded. Mean scoring was adopted. High scores of the three subscales separately reflect high level or severity of depression, anxiety, or stress. A previous study revealed that the Chinese version had good internal consistency and factor structure (Wang et al., 2016). The Cronbach's alpha of the whole scale for community sample is 0.85 and that of the student sample is 0.84.

### Data analysis plan

The community sample was used for EFA. The representation and generalization of the factor structure was guaranteed because both meditators and non-meditators were involved in the community sample. Regarding the issue of item parceling discussed in the Section Introduction, the item level of FFMQ was investigated. Baer et al. (2006) reported that Principal Axis Factoring with Direct Oblimin method was adopted. To retain or reject one item in the EFA, the following criteria adopted in previous practices (Tucker and Lewis, 1973; Marsh et al., 2005) were used: (a) items with communalities <0.30 were deleted, (b) factor loadings should be higher than 0.30, (c) items should not be highly cross-loaded, (d) items should be loaded on theoretically correct factors, (e) alpha coefficients for each subscale should be at least 0.70, and (f) items should possess acceptable model fit. A short form to reflect the essential facets of mindfulness was expected. Confirmatory factor analysis (CFA) was subsequently conducted using the student sample. The ML estimator was adopted for CFA because the five-point Likert scale can be considered a continuous variable (Prescott, 2004). Previous studies (e.g., Curtiss and Klemanski, 2014) indicated that three comparable models (i.e., One Single Factor Model, Related-factor Model, and Hierarchical-factor Model) were constructed to examine whether a hierarchical latent factor reflected the general mindfulness. Hu and Bentler (1999) explained that an efficient structural equation model should possess SRMR values below 0.08, TLI and CFI values above 0.95 or 0.90, and RMSEA values below 0.05 or at least below 0.08 (RMSEA values beyond 0.08 were considered unacceptable fit). Myers et al. (2011) suggested that a sample size larger than 300 (current sample size for CFA is 347) was adequate for the population model of CFA. Descriptive statistics and differences analyses were then conducted. The scores of mindfulness among meditators were expected to be higher than that of non-meditators. No difference in the scores of mindfulness was also expected among students between T1 and T2. Finally, correlation and regression analysis were performed. Both outcomes of mental health, including Flourishing, Depression, Anxiety, and Stress, at T1 and T2 were set as dependent variables in each regression equation, and the facets of mindfulness at T1 were set as independent variables. Step-wise method was adopted for involving or removing any predictors. The regression analysis examined whether the selected facets of mindfulness could, or which facet of mindfulness could, predict the mental health of students in 6 months.

Data were analyzed using SPSS 21.0 and Mplus 7.0.

## Results

### Item selection and factor structure

The Kaiser–Meyer–Olkin (0.839) and Bartlett's Test of Sphericity (4793.292, *p* < 0.001) indicated that the current community database was acceptable for factor analysis. Items were removed or retained on the basis of the proposed criterion in the Data Analysis Plan Section. First, 21 items had communalities less than 0.30 (e.g., Item 4 = 0.18). These items were removed individually from the lowest, which resulted in the removal of 24 items. The communalities of the 15 remaining items were above 0.30. Three factors (i.e., Describing, Act-aware, and Non-judging) were extracted to explain 62.64% of variances. Nevertheless, the factor structure and loadings were imperfect. For instance, Item 14 was cross-loaded on two factors with factor loadings of 0.49 and 0.44; Item 16 was cross-loaded on other two factors with loadings of 0.46 and 0.44. Items 14 and 16 were also incorrectly loaded on the Act-aware factor. Therefore, these two items were removed. The explained variances increased to 65.12%. Another round of inspection of items showed that Items 38 and 18 of Act-aware factor presented the same factor loadings (0.56), but Item 38 also loaded on other factors with loadings higher than 0.25. On the other hand, Item 18 had lower loadings on the other factors (0.09 and 0.06). Therefore, Item 38 was deleted. The final 12 items were consistent with those selected in previous studies (Tran et al., 2013, 2014; Hou et al., 2014).

**Figure 1:**
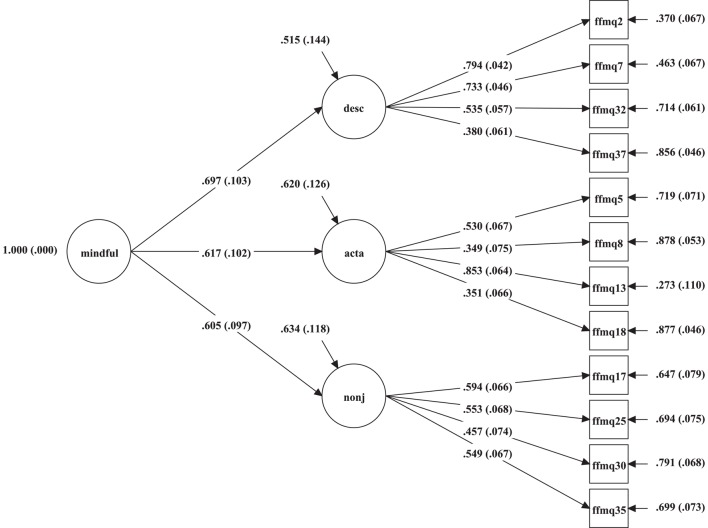
**Standardized path coefficients of the SIM-C model in student sample**. Mindful, Mindfulness; desc, Describing; acta, Act-Aware; nonj, Non-judging; All paths are significant at 0.001 level.

Table 1 provides the factor structure and loadings of the 12 remaining items, which had loadings higher than 0.46 and completely explained 67.56% of variances. This short form of FFMQ was renamed Short Inventory of Mindfulness Capability (SIM-C). The student sample was used to further investigate the factor structure of SIM-C through CFA. On the basis of previous studies, three comparable models were constructed, including the single factor model, related-factor model, and hierarchical-factor model. The results in Table 2 indicated that both related and hierarchical models had acceptable indices. Similar results were obtained in previous studies (Duan et al., 2012, 2013; Ho et al., 2016). Figure 1 illustrates the standardized path coefficients of the hierarchical-factor model.

**Table 1:**
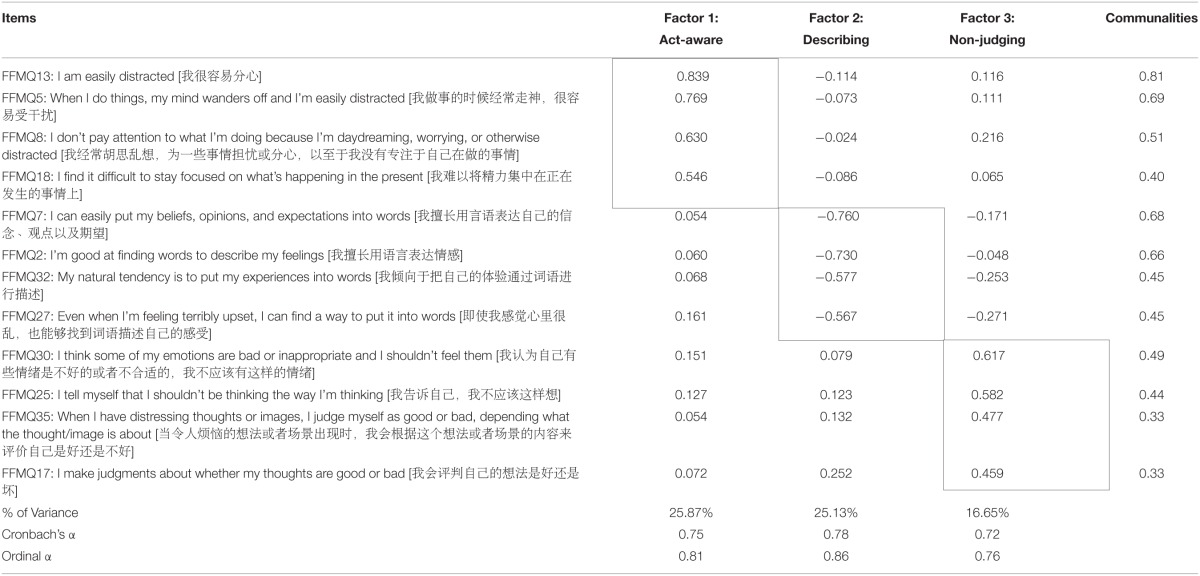
**Principal axis factoring with Direct Oblimin Method and loadings of the SIM-C**.

**Table 2:** **Goodness-of-fit indices for confirmatory factor analysis (student sample)**.

	**Goodness-of-fit Indices**				
	**CFI**	**TLI**	**SRMR**	**RMSEA**	**90% CI**
Single factor model	0.655	0.579	0.083	0.102	[0.088, 0.116]
Related-factor model	0.925	0.903	0.054	0.049	[0.030, 0.066]
Hierarchical-factor model	0.925	0.903	0.054	0.049	[0.030, 0.066]

### Reliability

Both traditional Cronbach's alpha and ordinal reliabilities (Gadermann et al., 2012) were computed. The internal consistency coefficients of the three subscales were satisfactory among the community and student populations. In the community sample, the Cronbach's alpha of the SIM-C is 0.82; that of the Observing, Act-aware, and Non-judging subscale is 0.75, 0.79, and 0.76, respectively. The ordinal reliabilities of the SIM-C is 0.84; that of the Observing, Act-aware, and Non-judging subscale is 0.80, 0.82, and 0.80, respectively. In the student sample (T1), the Cronbach's alpha of the SIM-C is 0.82; that of the Observing, Act-aware, and Non-judging subscale is 0.78, 0.80, and 0.74, respectively. The ordinal reliabilities of the SIM-C is 0.85; that of the Observing, Act-aware, and Non-judging subscale is 0.81, 0.85, and 0.78, respectively.

### Descriptive, correlation, and difference analyses

Table 3 presents the descriptive and differences results of the researched variables. In the community sample, participants with meditation experience showed higher scores of all three facets of mindfulness and total score of SIM-C than those without meditation experience. In the student sample, the total score of SIM-C at T2 is lower than that at T1. Nevertheless, the Describing subscale only exhibited a significant change, which decreased from 3.17 to 3.03 (*t* = 3.30, *p* < 0.05). The test–retest reliabilities of SIM-C and corresponding subscales among students are provided in Table 4; the values ranged from 0.61 to 0.68. Generally, the mindfulness and three facets are positive associated with Flourishing and negatively associated with Psychological Symptoms (i.e., Depression, Anxiety, and Stress). The correlation coefficients between T1 and T2 were weaker than the correlations of variables at the same time point (i.e., T1 or T2).

**Table 3:** **Descriptive and differences analyses of the SIM-C in different samples**.

	**Mindfulness**		**Describing**		**Act-aware**		**Non-judging**	
	**Mean (*SD*)**	***t*-test**	**Mean (*SD*)**	***t*-test**	**Mean (*SD*)**	***t*-test**	**Mean (*SD*)**	***t*-test**
Total community sample	2.99 (0.46)		3.36 (0.77)		3.14 (0.91)		2.47 (0.65)	
Non-meditator subsample	2.87 (0.42)	−6.19^**^	3.20 (0.76)	−4.87^**^	3.02 (0.88)	−2.85^*^	2.38 (0.61)	−3.19^*^
Meditator subsample	3.13 (0.47)		3.55 (0.73)		3.27 (0.91)		2.58 (0.68)	
Student sample_t1	3.24 (0.60)	2.57^*^	3.17 (0.67)	3.30^*^	3.08 (0.85)	1.23	3.46 (0.83)	0.90
Student sample_t2	3.16 (0.63)		3.03 (0.82)		3.02 (0.85)		3.42 (0.81)	

*t1, Time 1; t2, Time2*.

* p < 0.01;

** *p < 0.001*.

**Table 4:** **Correlation results of the mindfulness and other psychological constructs at two time points among student sample**.

	**1**	**2**	**3**	**4**	**5**	**6**	**7**	**8**	**9**	**10**	**11**	**12**	**13**	**14**	**15**
1 Minfulness_t1	–														
2 Describing_t1	0.71^**^	–													
3 Act-aware_t1	0.78^**^	0.36^**^	–												
4 Non-judging_t1	0.78^**^	0.36^**^	0.37^**^	–											
5 Minfulness_t2	0.68^**^	0.35^**^	0.48^**^	0.39^**^	–										
6 Describing_t2	0.39^**^	0.62^**^	0.24^**^	0.24^**^	0.77^**^	–									
7 Act-aware_t2	0.38^**^	0.17^**^	0.63^**^	0.25^**^	0.75^**^	0.36^**^	–								
8 Non-judging_t2	0.46^**^	0.19^**^	0.41^**^	0.61^**^	0.76^**^	0.41^**^	0.33^**^	–							
9 Flourishing_t1	0.36^**^	0.34^**^	0.30^**^	0.19^**^	0.36^**^	0.30^**^	0.28^**^	0.24^**^	–						
10 Flourishing_t2	0.21^**^	0.22^**^	0.18^**^	0.10	0.36^**^	0.35^**^	0.20^**^	0.28^**^	0.67^**^	–					
11 Depression_t1	−0.29^**^	−0.10	−0.41^**^	−0.13^*^	−0.21^**^	−0.06	−0.21^**^	−0.21^**^	−0.46^**^	−0.33^**^	–				
12 Depression_t2	−0.11	−0.01	−0.20^**^	−0.02	−0.12^*^	−0.08	−0.12^*^	−0.08	−0.29^**^	−0.43^**^	0.51^**^	–			
13 Anxiety_t1	−0.24^**^	−0.11^*^	−0.26^**^	−0.17^**^	−0.16^**^	−0.03	1.20	−0.13^*^	−0.26^**^	−0.16^**^	0.61^**^	0.31^**^	–		
14 Anxiety_t2	−0.17^**^	−0.09	−0.20^**^	−0.10	−0.07	0.02	−0.12^*^	−0.06	−0.22^**^	−0.21^**^	0.51^**^	0.50^**^	0.69^**^	–	
15 Stress_t1	−0.28^**^	−0.15^**^	−0.36^**^	−0.11^*^	−0.21^**^	−0.11^*^	−0.22^**^	−0.14^**^	−0.38^**^	−0.28^**^	0.27^**^	0.38^**^	0.68^**^	0.55^**^	–
16 Stress_t2	−0.12^*^	−0.05	−0.17^**^	−0.04	−0.05	−0.03	−0.17^**^	0.08	−0.22^**^	−0.30^**^	0.44^**^	0.64^**^	0.47^**^	0.59^**^	0.59^**^

*t1, Time 1; t2, Time 2*.

* p < 0.05;

** *p < 0.01*.

### Regression results of mindfulness in affecting mental health outcomes in student sample

To examine the predictive ability of SIM-C in affecting mental health outcomes, eight hierarchical regressions were set up with Flourishing, Depression, Anxiety, and Stress as the dependent variables at T1 and T2. At T1, both Describing and Act-aware facets significantly explained the 15% variance of Flourishing, which also applied to Flourishing at T2. Nevertheless, the explained variance in the latter was only 5%. For Anxiety and Stress at T1 and T2, Act-aware was the only facet that significantly explained variances. In the current sample, the three facets of mindfulness at T1 cannot predict depression symptoms at T2. Table 5 displayed the main results of regressions.

**Table 5:** **Regression results of the predictive ability of SIM-C on mental health outcomes in student sample**.

	**Time 1**							
	**Flourishing_t1**		**Depression_t1**		**Anxiety_t1**		**Stress_t1**	
	***B***	***t***	***B***	***t***	***B***	***t***	***B***	***t***
Describing_t1	0.37	5.01^**^	–	–	–	–	–	–
Act-aware_t1	0.23	3.91^**^	−0.26	−8.24^**^	−0.17	−4.98^**^	−0.25	−7.15^**^
Non-judging_t1	–	–	–	–	–	–	–	–
*R*^2^	0.15		0.16		0.07		0.13	
*F*	31.42^**^		67.87^**^		24.83^**^		51.15^**^	
	**Time 2**							
	**Flourishing_t2**		**Depression_t2**		**Anxiety_t2**		**Stress_t2**	
	***B***	***t***	***B***	***t***	***B***	***t***	***B***	***t***
Describing_t1	0.32	3.26^**^	–	–	–	–	–	–
Act-aware_t1	0.16	2.04^*^	−0.16	−3.86^**^	−0.14	−3.72^**^	−0.13	−3.20^*^
Non-judging_t1	–	–	–	–	–	–	–	–
*R*^2^	0.06		0.04		0.04		0.03	
*F* (sig.)	11.25^**^		14.90^**^		13.82^**^		10.23^*^	

*t1, Time 1; t2, Time 2*.

* p < 0.05;

** *p < 0.01*.

## Discussion

This study developed a SIM-C to measure the facets of mindfulness among community, including individuals with and without self-reported meditation experience, and student samples. The SIM-C was a 12-item self-reporting scale for assessing the Describing, Act-aware, and Non-judging facets of mindfulness (four items per facet). The psychometric evaluation demonstrated the acceptable factor structure of the measurement with good factor loadings, good internal consistency, and criterion-related validities as well as significant predictive validities. Generally, mindfulness capability was defined as the ability of using language to describe internal and external experiences, acting intentionally, and retaining non-judging attitude.

This study makes several contributions. First, the SIM-C was developed and validated at the item level of the original FFMQ. Previous studies (e.g., Baer et al., 2006; Deng et al., 2011; Curtiss and Klemanski, 2014; Hou et al., 2014; Williams et al., 2014) used item parceling to examine the psychometric properties of the FFMQ, which might overlook the content reflected by specific items. The present study retained the specific items for a better understanding of the latent factor of mindfulness and provided a foundation for measurement invariance tests in the future. Second, this study examined the capability facets of FFMQ in unspecific samples. Three facets, namely, Observing, Act-aware, and Non-judging, existed in adults with meditation experience, adults without meditation, and college students. The present-centered awareness/attention and non-judgmental attitude reflected by the three facets were a consensus reached by all existing definitions of mindfulness. Third, the concise SIM-C with good reliability, validity, and predictive ability was suitable for clinical participants, especially individuals with mental health issues (e.g., depression and anxiety disorder). The short form was particularly efficient for large-scale testing and intervention monitoring (Ziegler et al., 2014a,b).

One portrayal of mindfulness was consistent throughout its conceptualization in previous literature. Kabat-Zinn (1990) defined mindfulness as “the awareness that emerges through paying attention on purpose, in the present moment, and non-judgmentally to the unfolding of experience moment to moment.” Salzberg and Goldstein (2001) described mindfulness as a non-habitual state of being present during meditation. Brantley (2003) defined mindfulness as a friendly, non-judging, and present-moment awareness. Germer et al. (2005) defined mindfulness as the awareness and acceptance of the present experience. Baer (2003) recognized mindfulness as a psychological process and referred to it as “the non-judgmental observation of the ongoing stream of internal and external stimuli as they arise.” Bishop et al. (2004) further offered an operational definition of mindfulness: “self-regulation of attention so that it is maintained on immediate experience, thereby allowing for increased recognition of mental events in the present moment” and “adopting a particular orientation toward one's experience that is characterized by curiosity, openness, and acceptance.” All of these definitions emphasized two common capabilities in mindfulness, namely, present-centered awareness/attention and non-judgmental attitude. Present-centered awareness/attention means the attentive observation and awareness from moment to moment, including current feelings, thoughts, and behaviors. Attitude emphasizes the orientation that one holds toward personal experience. Mindfulness is characterized by a non-judgmental attitude as well as curiosity, openness, and acceptance to experience (Keng et al., 2011). Furthermore, both the attention and attitude facets of mindfulness are emphasized in the Mindfulness-Based Cognitive Therapy (MBCT) model (Segal et al., 2002; Barnhofer et al., 2009; Semple et al., 2010). MBCT was initially developed by combining the components of mindfulness with the cognitive model of vulnerability to depression (Segal et al., 2002). This model assumes that patterns of negative thinking (e.g., negative attention bias, rumination, and self-devaluation) initiate depression. MBCT recognizes that internal and external experiences, including internal thoughts and emotions as well as external body sensations, are simply phenomena that should be under *Being Aware* rather than *Judging* and should be *Describing* rather than *Changing* (Semple et al., 2010). Accordingly, the main principles of MBCT can change the cognitive pattern (i.e., attention and attitude) through several mindfulness-related training or skills. Therefore, the conclusion that “present-centered awareness/attention” and “non-judgmental attitude” are the two commonly accepted facets of mindfulness is reasonable (Bishop et al., 2004; Lau et al., 2006; Quaglia et al., 2014).

Among the five original facets of FFMQ, Observing, Describing, and Act-aware clearly have close relationships with “present-centered awareness/attention,” and Non-judging and Non-react have close relationships with “non-judgmental attitude.” The SIM-C comprised Describing (using words to identify and express internal experience), Act-aware (paying full attention to the current activities without an automatic pilot), and Non-judging (non-evaluation of internal experiences and stimuli) facets and excluded two other facets originally involved in FFMQ (i.e., Observing or “noting both internal and external stimuli” and Non-react or “no action toward emerging internal experience”). This result was expected on the basis of the literature review. Given the phrasing issues and poor psychometric properties of items in Non-react, its exclusion was not surprising. However, Lilja et al. (2013) indicated that Observing was an essential facet in mindfulness and compared the different patterns of Observing facet among meditators and non-meditators. The results showed that the level of Observing consistently determined the level of mindfulness regardless whether the participants were meditators or non-meditators. Nevertheless, explaining why Observing has opposite associations with psychological distress among meditators and non-meditators is difficult under this interpretation. We argued that Lilja et al. (2013) theoretically hypothesized Observing as a facet of mindfulness and further explored the significances of Observing, thereby ignoring the fundamental issue, that is, the differences between dispositional and cultivated mindfulness. Baer et al. (2008) previously suggested that Observing might indicate the tendency of rumination. One recent controlled randomized clinical trial indicated that mindfulness-based interventions have null to weak effects (*d* = 0.15–0.32) in improving the Observing and Non-react facets of mindfulness (i.e., dispositional mindfulness); nevertheless, the three other facets (i.e., Describing, Act-Aware, and Non-judging; cultivated mindfulness) were significantly enhanced (*d* = 0.29–0.63) (Goldberg et al., 2016). Goldberg et al. (2016) further implied that the current version of FFMQ “does not truly measure the construct of mindfulness, despite the construct validity previously reported for this measure.” Another community-based mindfulness training found that the awareness and non-judgmental component of mindfulness among 90 participants in the experimental group was significantly improved (Szekeres and Wertheim, 2015). Therefore, empirical evidence implied that the Describing, Act-aware, and Non-judging facets should comprise the capability facet of mindfulness in the present research area.

The current study established both the correlated and hierarchical factor structures of mindfulness capability. The identical goodness-of-fit of the two models suggested that the three facets of mindfulness shared many variances at a global level. Similar results were found in previous studies on character strengths (Duan et al., 2012, 2013; Duan and Ho, 2016; Ho et al., 2016). Accordingly, a higher-order factor is the better choice for most research or practice that does not need to examine the role of each facet. However, researchers who intend to study the specific role or efficacy of each facet should calculate the scores of different facets. For instance, Peters et al. (2013) found an interaction effect between awareness-based (i.e., Act-aware) and non-judging-based (i.e., Non-judging) skills and further indicated that a less judgmental stance benefited acting with awareness in borderline personality disorder patients.

Certain limitations should be mentioned. First, the participants in communities were self-identified meditators or individuals with meditation experience. Most of them did not attend any official mindfulness training. Thus, the meditation experience might be different, and this difference might be neglected in previous studies. Future studies must screen the true meditators. The SIM-C should be validated among experienced meditators; Years of Meditation and Average Meditation Time per Week should be measured. Second, previous studies conducted in Western countries indicated that the hierarchical model, regardless whether four factor or five factor among meditators or non-meditators, was better than the correlated model (Baer et al., 2008; Curtiss and Klemanski, 2014; Williams et al., 2014). However, the studies administrated in Eastern countries found that the correlated model was better than the hierarchical model (Deng et al., 2011; Hou et al., 2014). Future studies should investigate cultural differences. Moreover, advanced statistics and models such as Latent State-Trait Model should be adopted to validate the differences between dispositional and cultivated mindfulness. The Latent State-Trait Model systematically considers the method factors, situations of measurement and the person-situation interactions, which will show more evidences to the measurements of mindfulness. Finally, the SIM-C should be applied to an intervention study for monitoring the change process of mindfulness and the change of mental well-being. The newly developed instrument will provide additional solid measurement–sensitive evidence.

## Author contributions

WD conceived and designed the experiments, performed the experiments, analyzed the data, contributed reagents/materials/analysis tools, data collection, wrote the paper, prepared figures and/or tables, reviewed drafts of the paper. JL helped to prepare figures and/or tables and review the comments of the paper.

## Funding

The author(s) disclosed receipt of the following financial support for the research, authorship, and/or publication of this article: Wuhan University Humanities and Social Sciences Academic Development Program for Young Scholars “Sociology of Happiness and Positive Education.”

### Conflict of interest statement

The authors declare that the research was conducted in the absence of any commercial or financial relationships that could be construed as a potential conflict of interest.
